# Fracture behaviour of historic and new oak wood

**DOI:** 10.1007/s00226-018-1038-6

**Published:** 2018-07-18

**Authors:** R. A. Luimes, A. S. J. Suiker, C. V. Verhoosel, A. J. M. Jorissen, H. L. Schellen

**Affiliations:** Eindhoven University of Technology, P.O. Box 513, 5600 MB Eindhoven, The Netherlands

## Abstract

Recent museum studies have indicated the appearance of cracks and dimensional changes on decorated oak panels in historical Dutch cabinets and panel paintings. A thorough analysis of these damage mechanisms is needed to obtain a comprehensive understanding of the causes of damage and to advise museums on future sustainable preservation strategies and rational guidelines for indoor climate specifications. For this purpose, a combined experimental-numerical characterization of the fracture behaviour of oak wood of various ages is presented in this communication. Three-point bending tests were performed on historical samples dated 1300 and 1668 A.D. and on new samples. The measured failure responses and fracture paths are compared against numerical results computed with a finite element model. The discrete fracture behaviour is accurately simulated by using a robust interface damage model in combination with a dissipation-based path-following technique. The results indicate that the samples dated 1300 A.D. show a quasi-brittle fracture response, while the samples dated 1668 A.D. and the new samples show a rather brittle failure response. Further, the local tensile strength of the oak wood decreases with age in an approximately linear fashion, thus indicating a so-called ageing effect. Numerical simulations show that, due to small imperfections at the notch tip of the specimen, the maximal load carrying capacity under three-point bending may decrease by maximally $7 \%$. A comparison between a calibration of the experimental results by isotropic and orthotropic elastic models shows that the peak load is 10–$13\%$ higher for the orthotropic elastic model. Finally, no significant dependence of the fracture toughness on the age of the oak wood and on the orientation of the fracture plane has been found. The strength and toughness values measured can be used as input for advanced numerical simulations on climate-induced damage in decorated oak wooden panels and panel paintings.

## Introduction

Decorated oak wooden panels in historical Dutch cabinets and panel paintings occasionally show damage that might originate from thermal and/or hygral climate fluctuations. To safely preserve these highly valuable and susceptible museum objects for future generations, preeminent museums apply severe preservation strategies by imposing strong limitations on indoor temperature and relative humidity fluctuations. This results in a low-risk preservation of susceptible museum objects, though at the expense of high energy demands and costs of large heating, ventilating and air conditioning (HVAC) systems. The understanding of the origin of damage and the possible harmful effects of climate fluctuations on museum objects is essential for improving future preservation strategies and sustainable guidelines for indoor climate specifications. Recently, decorated oak wooden panels were extensively analysed to characterize these objects in full detail (Ekelund et al. 2017; Ekelund and Jorissen 2014). Relevant empirical data, including aspects like construction category and type, panel and board dimensions, conservation treatments and damage occurrence, were collected from a large group of naturally aged museum objects consisting of 138 doors of 70 Dutch cabinets ranging from the sixteenth to the twentieth century, and 249 Dutch panel paintings produced between 1625 and 1690 made by artists born between 1601 and 1620. All panels were composed of high quality, radially cut oak wooden boards, where the veneer or marquetry layers were made of a variety of wood species or a combination of ground and paint layers. It was observed that shrinkage of approximately 1% of the original panel width was dominant in the radial material direction, and that visible cracks, when present, were mainly located along the wood grain direction. In addition, substantial glue-joint failure was observed at joints between the oak wood boards.

Advanced numerical models that can accurately simulate climate-induced damage development in decorated oak wooden panels, in combination with experimental tests and microscopy analyses of the oak wood cellular microstructure, can further improve the understanding and interpretation of the in situ observations mentioned above. The present paper presents a first step into this direction by focussing on the experimental-numerical characterization of the fracture behaviour of historic and new oak wood induced by mechanical loading. Three-point bending tests were performed to analyse the failure response and corresponding fracture path. Scanning electron microscopy was used to study the microstructure at the fracture plane. The modelling of discrete fracture patterns was carried out by using the finite element method (FEM), in which the continuum elements were surrounded by interface elements equipped with the mixed-mode interface damage model presented in Cid Alfaro et al. (2009). This approach was originally proposed in Xu and Needleman (1994), and allows for the description of complex cracking paths in arbitrary directions, including the effects of crack bifurcation and crack coalescence. Successful applications of this technique refer to, among others, the simulation of crazing in polymers (Tijssens et al. 2001) and the combined fracture and decohesion behaviour of fibrous composites (Cid Alfaro et al. 2010a, b). The dissipation-based path-following method presented in Gutiérrez (2004), Verhoosel et al. (2008) was implemented in the finite element model to allow for the robust simulation of brittle fracture responses with snap-back behaviour. To the best of the authors’ knowledge, this modelling strategy has not been used previously for the simulation of discrete fracture behaviour in wood; instead various other strategies were applied, see Landis et al. (2002), Qiu et al. (2014), Saft and Kaliske (2013), Lukacevic et al. (2015), Lukacevic and Füssl (2016).

The paper is organized as follows: The “Experimental program” section describes the test samples and the experimental setup for the three-point bending tests. The “Experimental results” section discusses the results of the experimental tests in terms of the load-deflection response, the fracture path and fracture toughness. The discrete fracture model is described in the “Numerical model” section. The numerical results are discussed in “Numerical results” and the  “Conclusion” section summarizes the main conclusions of the experimental-numerical study.

## Experimental program

**Fig. 1:**
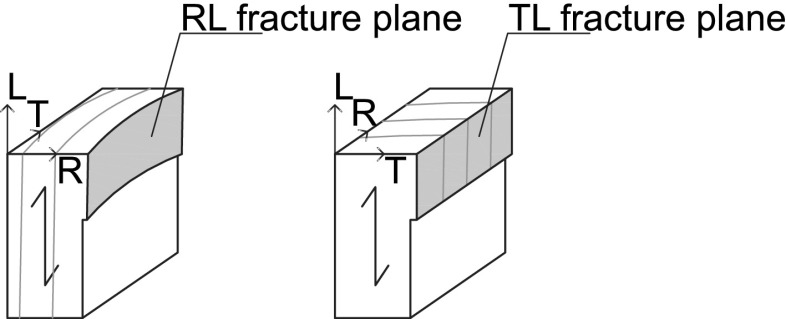
RL and TL fracture planes. The longitudinal, radial and tangential directions are indicated by the letters *L*, *R* and *T*, respectively

A recent museum study performed on a large group of panel paintings and decorated oak wooden panels in historical cabinets showed the presence of visible cracks that were predominantly located along the wood grain direction (Ekelund et al. 2017; Ekelund and Jorissen 2014). For the understanding of the origin of these cracks, the fracture characteristics of oak wood as a function of age need to be determined, which is done by analysing the failure response of historic and new oak wood under three-point bending. In the experimental setup, the material orientation of the oak wood specimens is such that the expected catastrophic failure crack runs along the wood grain direction (i.e. the longitudinal direction), with the crack plane normal either oriented in the tangential direction (a TL crack) or in the radial direction (an RL crack), see Fig. 1.

### Test samples

**Fig. 2:**
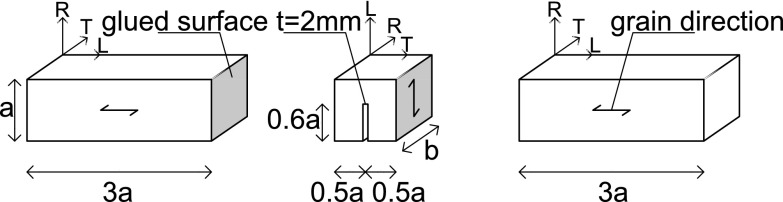
Geometry and dimensions of the test specimens. The specimen is composed of a central square block with a notch, which is either made of historical oak wood or new oak wood, and is sandwiched by two supporting beams made of new oak wood. The longitudinal, radial and tangential directions are indicated by the letters *L*, *R* and *T*, respectively. The specimen as illustrated in the figure can be used to develop a TL crack; for growing an RL crack the tangential and radial directions indicated for the central block need to be switched

In accordance with Nordtest (1993), the test specimens were composed of two supporting beams with dimensions $3a \times a \times b$ sandwiching a square block of $a \times a \times b$, see Fig. 2. Two different specimen sizes were selected that are representative of the dimensions of museum objects (Ekelund et al. 2017), namely a relatively *large specimen* with $a=45$ mm and $b=30$ mm and a relatively *small specimen* with $a=25$ mm and $b=20$ mm. The bond between the square block and the supporting beams was realized by using a PVA glue (NOVA COL D2 A). The square block was equipped with a central notch of length 0.6*a* and thickness $t=2$ mm to trigger the onset of fracture at the mid-span and to ensure crack propagation at the centre of the specimen along the TL or RL material directions. The supporting beams were made of new oak wood and the square block at the centre of the specimen was made of either historic oak wood, dated 1300 or 1668 A.D., or new oak wood. The specimens of a specific age were made from one wood sample. The historic oak wood was provided by the Agency for Palaces and Cultural Properties in Denmark and the National Museum of Denmark. Archival sources and building archaeology were considered to determine the date of origin. For the specimens containing a historic oak wooden square block with a TL crack, both large and small specimens were tested, and for the specimens containing a historic oak wooden square block with an RL crack, only a small specimen was tested. For the specimens completely composed of new oak wood and containing a TL or RL crack, both large and small specimens were tested, see Table 1 for an overview. Each specific specimen configuration was tested 5–7 times in order to verify the repeatability of the experiment and to determine the spread in the test results.

### Sample preparation and experimental setup

Prior to testing, all specimens were prepared in accordance with the following step-wise procedure. First, the beams and square block were conditioned in a climate chamber at a temperature of $20\,^\circ$C and a relative humidity of 60%. When the equilibrium moisture content (EMC) was reached (at which the sample weight remains constant over time), the components were bonded together with PVA glue. After the curing process of the glue was finished, the test specimen was taken out of the climate chamber. Subsequently, a central notch of 0.6*a* was created using a blunt saw and a file, after which a three-point bending test was performed. When the test was finished, the EMC of the square block was determined for which the mass at 60% relative humidity and the oven dry mass were measured of a small part taken from the square block, in accordance with the procedure described in ISO (1975). The average EMC for all specimen configurations ranged between 10.4 and 15.0%.

**Fig. 3:**
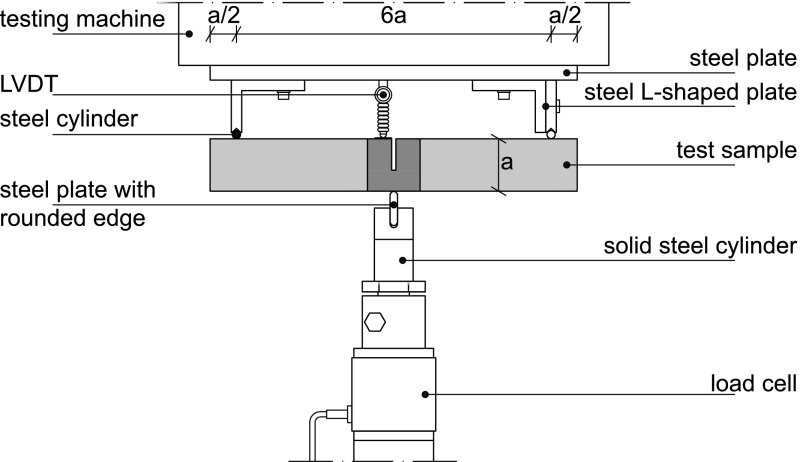
Experimental setup of an oak wood specimen subjected to three-point bending

The experimental setup for the three-point bending test shown in Fig. 3 meets the requirements described in Nordtest (1993). The loading apparatus used is an Instron 5985, equipped with a 5 kN load cell. The test specimen was simply supported at a span $L=6a$, where the supports were composed of a solid steel L-shaped plate and a solid steel cylinder. A quasi-static load was applied in a displacement-controlled fashion at the mid-span of the specimen via a steel plate with a rounded edge. For the small and large test samples, the loading rate was equal to 0.4 and 0.6 mm/min, respectively. The magnitude of the load was measured by a load cell and the load point deflection was monitored through a linear variable displacement transformer (LVDT) at the mid-span. The fracture toughness $G_\mathrm{c}$ of the test specimen is characterized by the area under the load–displacement curve divided by an anticipated, straight fracture surface above the notch of $A=0.4ab$, i.e.

1 $$\begin{aligned} G_\mathrm{c} \, = \, \frac{1}{A}\int _{0}^{w}\hat{F}\left( w\right) dw, \end{aligned}$$

where $F=\hat{F}(w)$ is the applied load and *w* is the load point deflection.

## Experimental results

### Load-deflection response

**Fig. 4:**
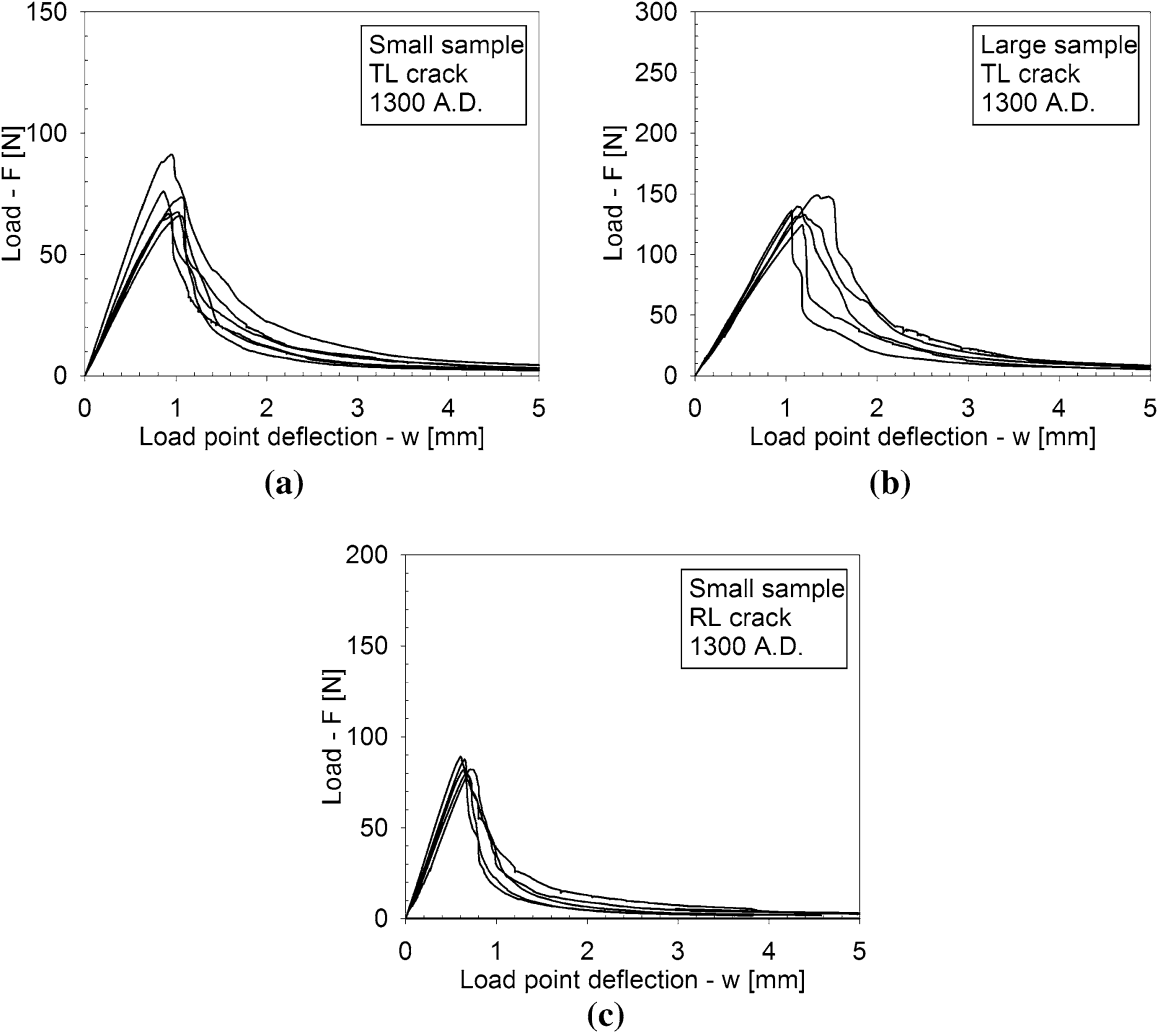
Experimental load–displacement curves for historic oak wooden beams dated 1300 A.D. under 3-point bending. **a** Small sample with a TL crack. **b** Large sample with a TL crack. **c** Small sample with an RL crack. Note that the scales used on the vertical axes of the three figures are different, which is done for an adequate comparison of the results with those of the oak wood dated 1668 A.D. and the new oak wood, see Figs. 5 and 6, respectively

**Fig. 5:**
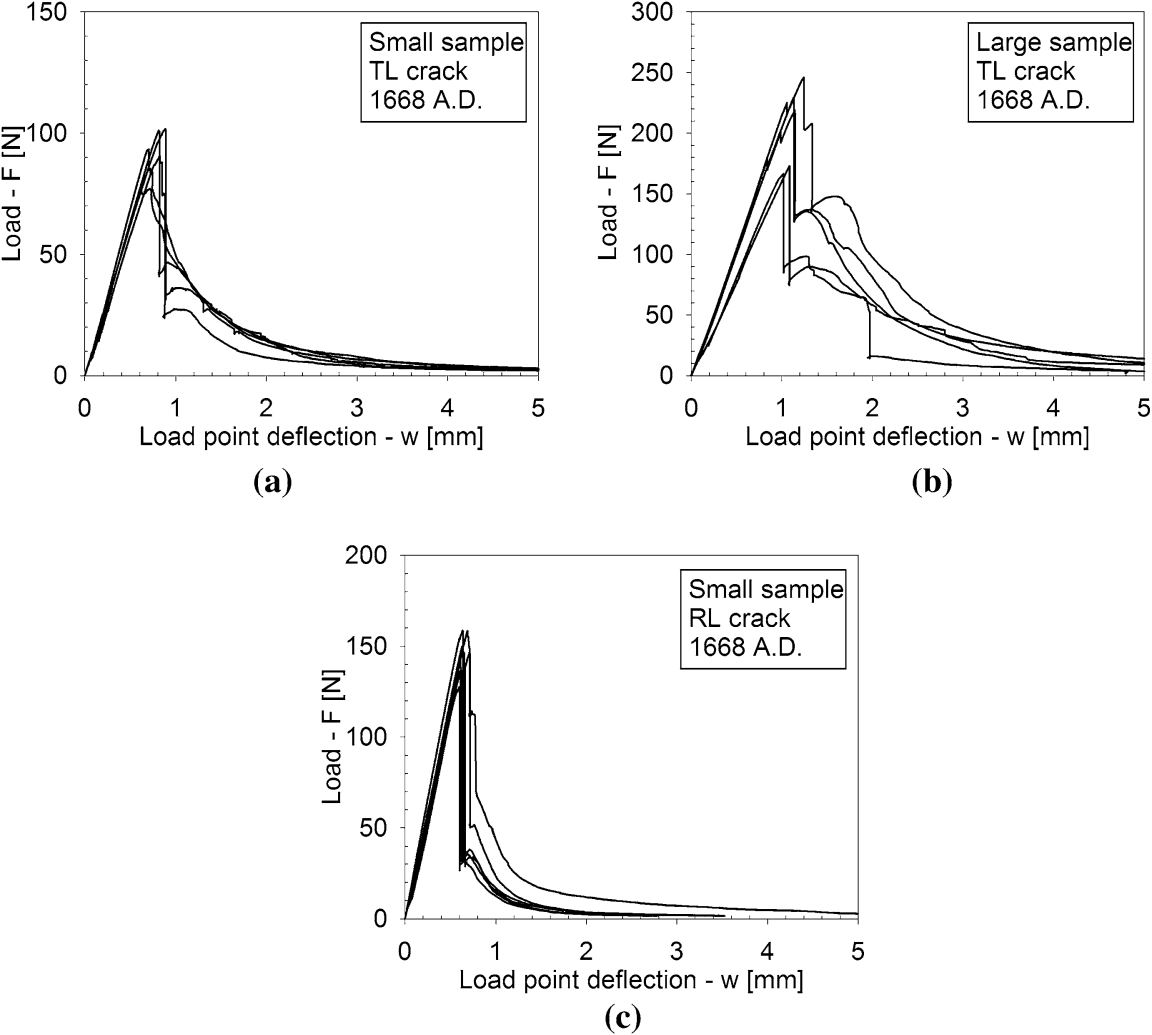
Experimental load–displacement curves for historic oak wooden beams dated 1668 A.D. under 3-point bending. **a** Small sample with a TL crack. **b** Large sample with a TL crack. **c** Small sample with an RL crack

**Fig. 6:**
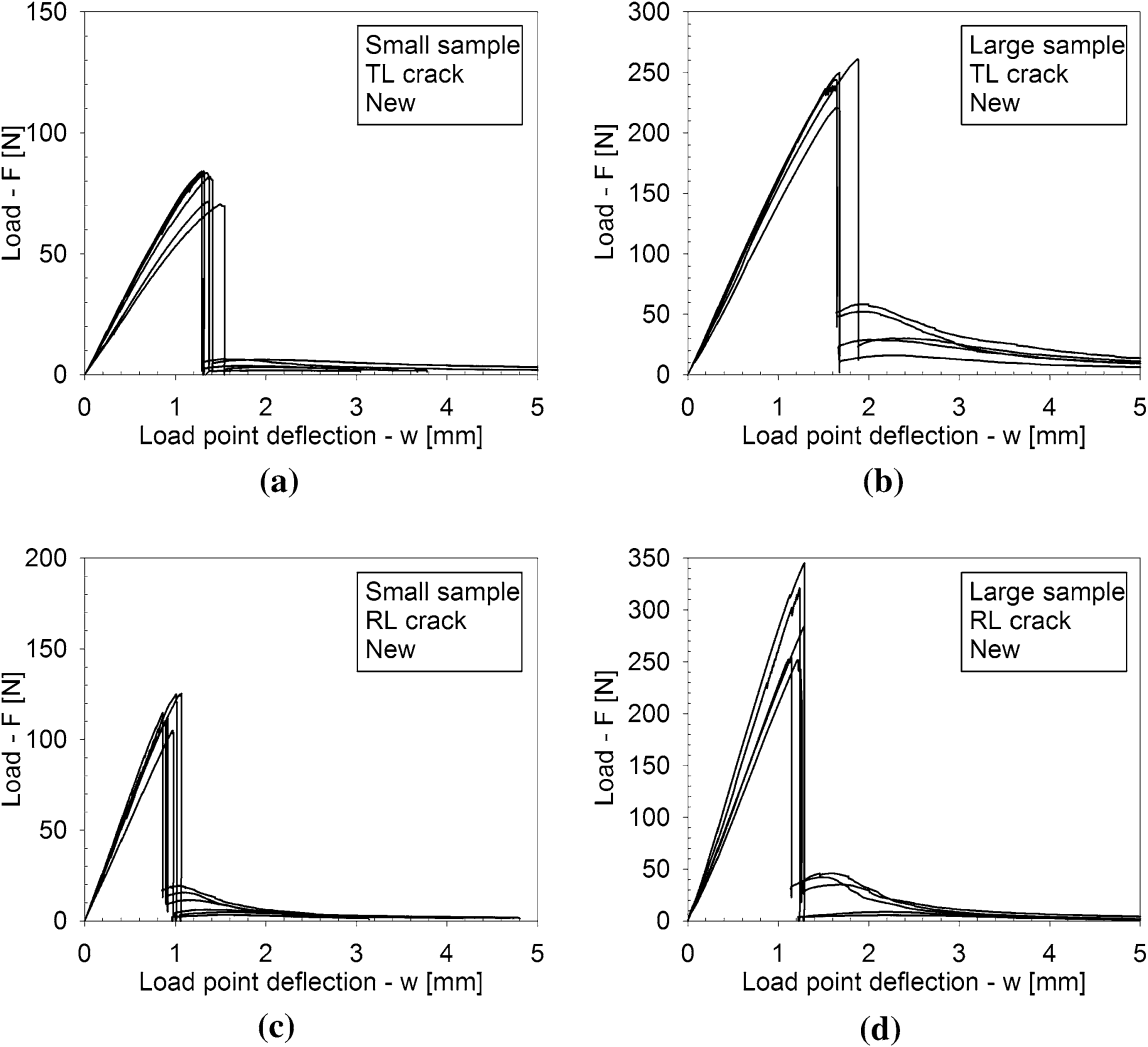
Experimental load–displacement curves for new oak wooden beams under 3-point bending. **a** Small sample with a TL crack. **b** Large sample with a TL crack. **c** Small sample with an RL crack. **d** Large sample with an RL crack

Figures 4, 5 and 6 depict the applied load *F* versus the load point deflection *w* for, respectively, the historic oak wood dated 1300 and 1668 A.D. and the new oak wood, with figures (a), (b) and (c) illustrating the responses for the TL crack in the small and large specimens, and the RL crack in the small specimen, respectively. For all specimens, the load initially increases approximately linearly with increasing displacement, representing an elastic response. Close to the peak strength, the failure process initiates through the nucleation of micro-cracks. When these micro-cracks start to coalesce, the peak strength is reached, after which clear differences appear in the failure responses of the historic and new samples. For the historic samples dated 1300 A.D., the load monotonically decreases with increasing displacement, in correspondence with the development of a macroscopic failure crack. This softening process continues until the load has dropped to zero and the failure crack has fully mobilized across the complete specimen height. Conversely, for the historical samples dated 1668 A.D., a sudden *dynamic* drop in load can be observed, during which the load point deflection remains constant. As will be demonstrated by the numerical analyses in the  “Numerical results” section, this drop in load occurs since the actual quasi-static response here would be characterized by a so-called *snap-back behaviour*, whereby a *decrease* in load magnitude is accompanied by a *decrease* in load point deflection. This essentially indicates that the energy incrementally dissipated by the relatively brittle failure crack is lower than the energy incrementally released due to elastic unloading of the rest of the specimen. Consequently, the snap-back behaviour may be caused by (i) a relatively low fracture toughness, and/or (ii) a relatively high specimen stiffness. In the “Numerical results” section, these two effects are explicitly distinguished for the three different age categories of the oak wood samples, by mimicking their experimental fracture response with advanced finite element simulations. Since the three-point bending test is performed in a displacement-controlled fashion, the snap-back behaviour cannot be followed experimentally, and therefore results in an abrupt dynamic decrease in load magnitude. Observe though, that in most cases the measured load signal does not straightforwardly drop to zero; instead, it picks up the quasi-static failure response at a relatively low load level and continues to follow the corresponding softening branch under increasing load point deflection, until the specimen is completely broken. The failure responses of the new samples and the historic samples dated 1668 A.D. in this sense are qualitatively comparable, although for the new samples the abrupt dynamic drop in load is somewhat larger.

### Fracture path

**Fig. 7:**
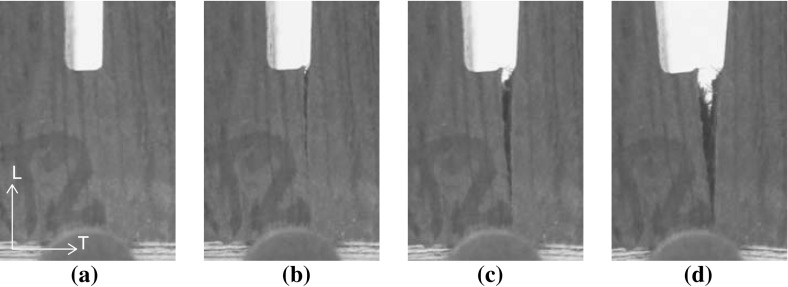
Fracture path of a small historical test sample dated 1300 A.D. with a TL crack. **a** Elastic response. **b** Crack initiation. **c** Crack propagation. **d** Ultimate failure

**Fig. 8:**
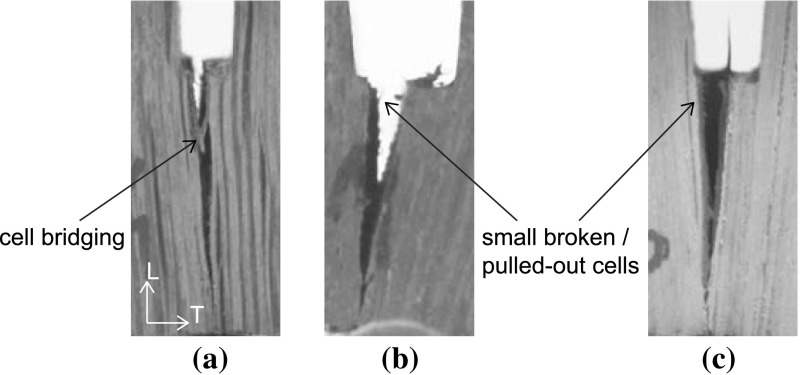
Fracture path at ultimate failure. **a** Historical sample dated 1300 A.D. **b** Historical sample dated 1668 A.D. **c** New sample

The fracture path of the small sample dated 1300 A.D. that fails by a TL crack is illustrated in Fig. 7 at various stages during the test procedure. After an initial, elastic response of the specimen (Fig. 7a), the crack nucleates at the right side of the notch (Fig. 7b), and subsequently develops along an approximately straight trajectory (Fig. 7c). Hence, the fracture path is in close correspondence with the ideally straight trajectory assumed for computing the fracture toughness $G_\mathrm{c}$ in Eq. (1). The resistance against fracture is governed by cell material bridging the crack faces, which, once broken, delaminated or pulled-out, preludes catastrophic failure of the specimen (Fig. 7d). In comparison with the historic specimen dated 1668 A.D. and the new specimen, the bridging of cell material in the oldest specimen is more prominent, see Fig. 8, which suggests that the failure response is less brittle than the failure responses of the historical specimen dated 1668 A.D. and the new specimen, see Figs. 4, 5 and 6.

**Fig. 9:**
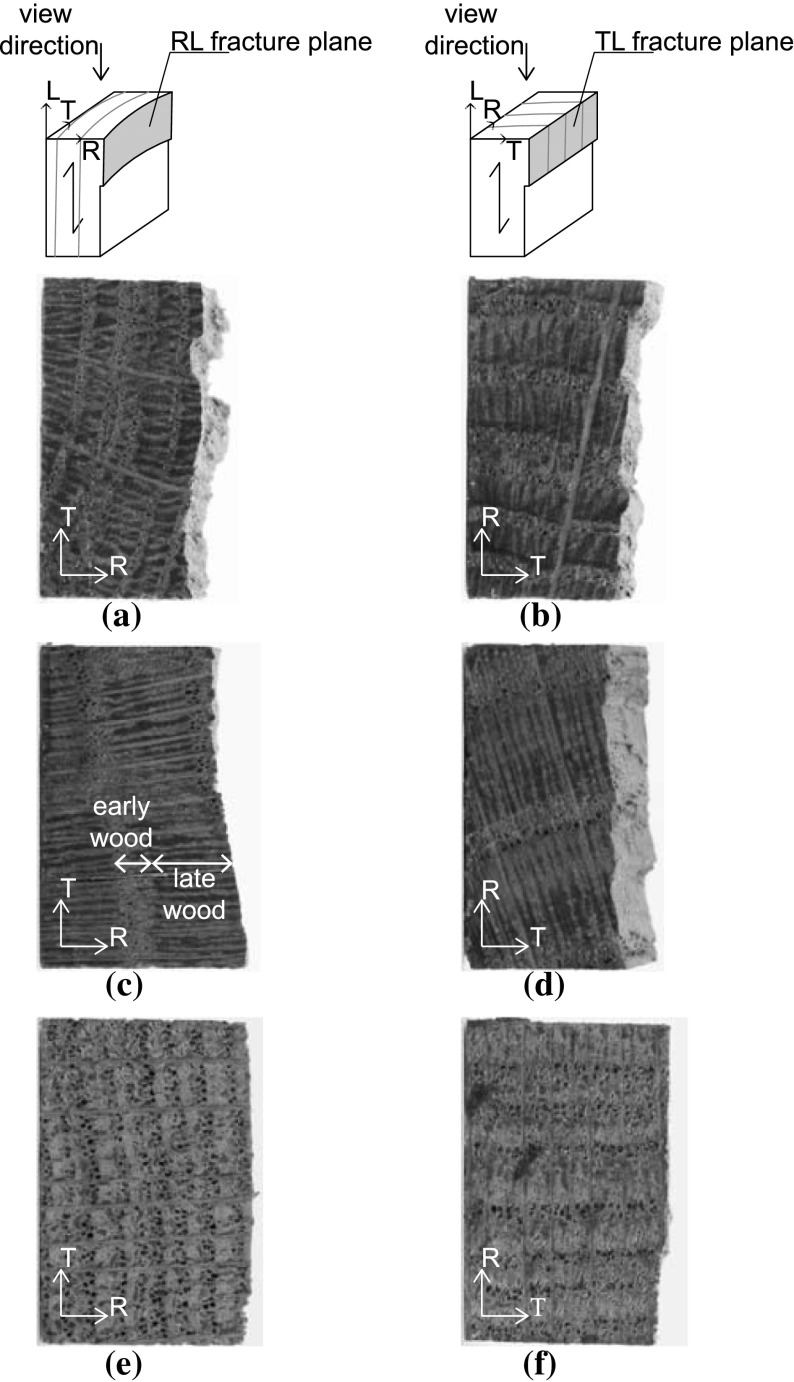
Top view of fracture plane. **a** Historical sample dated 1300 A.D. with an RL crack. **b** Historical sample dated 1300 A.D. with a TL crack. **c** Historical sample dated 1668 A.D. with an RL crack. **d** Historical sample dated 1668 A.D. with a TL crack. **e** New sample with an RL crack. **f** New sample with a TL crack

Another reason for differences in the brittleness of the failure responses is the small-scale geometrical features of the crack trajectory. As can be observed from Fig. 9, the crack faces are characterized by undulations, caused by differences in material density originating from the oak wood growth process. The oak wood of a particular age has a distinct microstructure with a specific colour, which is dark brown, brown and light brown for the historical specimens aged 1300, 1668 A.D. and the new specimen, respectively. The cross sections are characterized by the presence of 2–8 annual rings, whereby an annual ring is composed of a low density, early wood part and a high density, late wood part. The early wood consists of thin-walled fibres and large thin-walled vessels, while the late wood is determined by thick-walled fibres and small thick-walled vessels. Although care has been taken to align the specimen height and width with the RL and TL material directions of the wood microstructures, due to the relatively strong curvature of the annual rings some variation in material direction along the specimen height and width could not be avoided. These variations are different for the 5–7 specimens tested for each age category, which, as discussed below, may cause deviations in the fracture properties measured. The crack typically has a preference to cross the weaker, early wood. However, it occasionally has to traverse small sections of late wood in order to continue its propagation through a new section of early wood, thereby causing undulations at the fracture planes. The crack face undulations clearly are most pronounced for the historical samples dated 1300 A.D., see Fig. 9a for the RL crack and Fig. 9b for the TL crack, which corresponds to a relatively large fracture surface that makes the overall failure response less brittle, as can be observed by comparing Fig. 4 with Figs. 5 and 6. Note finally from Fig. 8 that the actual location of the initial crack may vary, since small geometrical irregularities at the notch tip trigger the specific location of crack initiation. This effect will be investigated in more detail in the “Numerical results” section when a comparison is made with the results from a detailed finite element study.

### Oak wood microstructure and fracture toughness

**Fig. 10:**
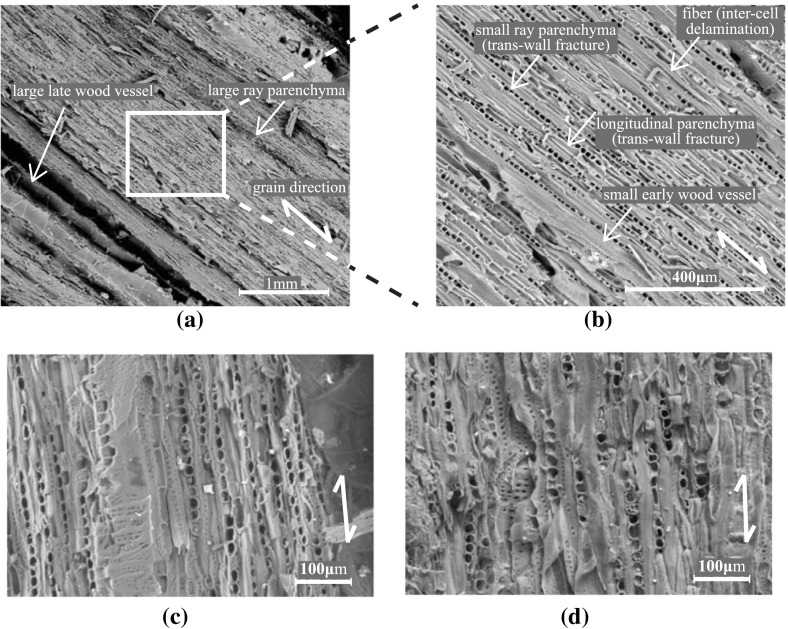
Microstructure at the RL fracture plane. **a** Historical sample dated 1300 A.D.; $\times$ 34 magnification, **b** Historical sample dated 1300 A.D.; $\times$ 131 magnification. **c** Historical sample dated 1668 A.D.; $\times$ 200 magnification. **d** New sample; $\times$ 200 magnification

**Fig. 11:**
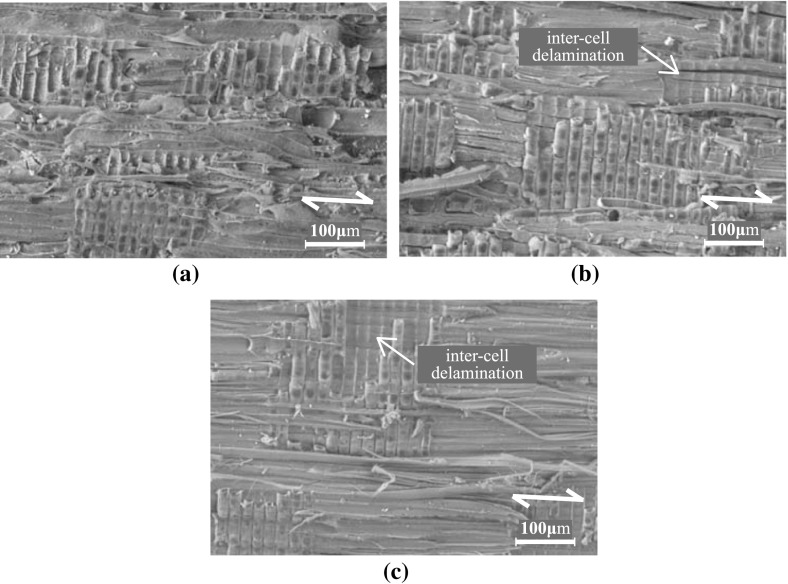
Microstructure at the TL fracture plane. **a** Historical sample dated 1300 A.D.; $\times$ 200 magnification. **b** Historical sample dated 1668 A.D.; $\times$ 200 magnification. **c** New sample; $\times$ 200 magnification

The microstructures of the fracture planes of small historical and new oak wood samples are represented in the scanning electron microscopy photographs in Figs. 10 and 11. Typical oak wood cell types can be observed, such as large early wood vessels, small late wood vessels, vessel tracheids, longitudinal parenchyma cells, horizontal small and large parenchyma ray cells, thinner-walled early wood fibres and thick-walled late wood fibres. For the historical wood, the number of rays is larger than for the new wood. Further, the historical wood has a lower number of fibres and longitudinal parenchyma cells. The vessels and parenchyma cells show mainly transwall fracture, i.e. broken cells, and the fibres show intercell failure, i.e. delamination. In addition, at the TL fracture plane, intercell delamination can be observed along the interfaces between rays and fibres and/or rays and longitudinal parenchyma cells.

**Fig. 12:**
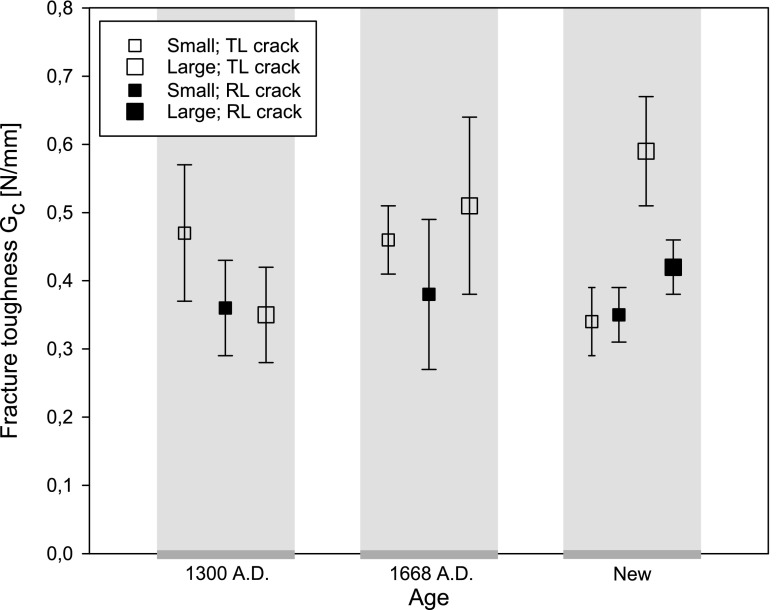
Fracture toughness $G_\mathrm{c}$ as determined via Eq. (1) from 5 to 7 tests. The square symbols indicate the average value and the error bars represent one standard deviation of uncertainty

From the load–displacement curves in Figs. 4, 5 and 6, the fracture toughness $G_\mathrm{c}$ is calculated in accordance with Eq. (1). In correspondence with the discussion in the “Load-deflection response” section, Eq. (1) is strictly valid if the softening branch of the experimental load–displacement curve is obtained in a quasi-static fashion, and thus is free from dynamic load drop events. Note that this is not the case for the historical specimens dated 1668 A.D. and the new specimens, whereby the toughness value computed with Eq. (1) thus partly loses its physical significance. In those cases, it merely serves as a global estimate, to which the toughness value systematically deduced from advanced finite element modelling will be compared, see the “Numerical results” section. The average value and the standard deviation of the fracture toughness, as determined with Eq. (1) from 5 to 7 tests results, are listed in Table 1. In Fig. 12 the average value of the fracture toughness is graphically represented, with the error bar indicating one standard deviation of uncertainty. Due to the significant spread in values, it is not possible to deduce clear differences between the toughnesses related to fracture along the various grain orientations. Also, no clear trend as a function of age can be deduced. The spread in toughness values is likely to originate from the heterogeneity of the microstructure, see Fig. 9, which showed to have some variations among the 5–7 specimens selected. Note that the average values of the fracture toughness approximately lie between 0.35 and 0.60 N/mm$^1$. The range in values stems from differences in the place of origin of the specific oak wood, the forest density, the local climate conditions, the climate history, etc.

The calibration procedure of the fracture toughness can be refined by accounting for the material anisotropy of oak wood, for the dynamic load drops observed in the responses of the historical oak wood dated 1668 A.D. and the new oak wood, and for the presence of imperfections at the notch tip. In addition, the local tensile strength of the oak wood samples needs to be determined. These aspects, however, ask for the accurate determination of the heterogeneous stress distribution in the specimens tested, which can be accomplished by means of dedicated finite element simulations that are discussed in the “Numerical model” and “Numerical results” sections.

**Table 1:** Fracture toughness $G_\mathrm{c}$ derived from the experiments using Eq. (1). The parameters $\mu _{G_\mathrm{c}}$ and $\sigma _{G_\mathrm{c}}$ represent the average value and the standard deviation, respectively

Date	Size	Crack orientation	$G_\mathrm{c}$ ($\text {N/mm}^1$)	
			$\mu _{G_\mathrm{c}}$	$\sigma _{G_\mathrm{c}}$
Historical 1300 A.D.	Small	TL crack	0.47	0.10
		RL crack	0.36	0.07
	Large	TL crack	0.35	0.07
Historical 1668 A.D.	Small	TL crack	0.46	0.05
		RL crack	0.38	0.11
	Large	TL crack	0.51	0.13
New	Small	TL crack	0.34	0.05
		RL crack	0.35	0.04
	Large	TL crack	0.59	0.08
		RL crack	0.42	0.04

## Numerical model

In the finite element model, the discrete fracture response measured experimentally is simulated by interface elements equipped with the mixed-mode damage model presented in Cid Alfaro et al. (2009). The brittle snap-back behaviour characteristic of historical oak wood dated 1668 A.D. and new oak wood is simulated by adopting the dissipation-based arc-length method presented in Gutiérrez (2004), Verhoosel et al. (2008). These aspects are outlined below, together with the definition of the geometry, material properties, boundary conditions, and spatial discretization characteristics.

### Geometry and boundary conditions

**Fig. 13:**
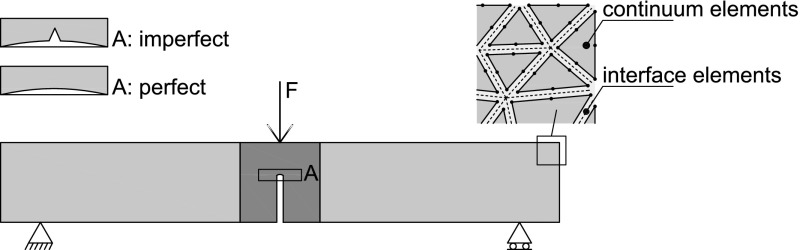
Geometry and boundary conditions of the test specimen and finite element discretization. Specimens with and without an imperfection at the notch tip are considered (inset left). The discrete fracture response is simulated by surrounding the continuum elements by interface elements (inset right)

The oak wood specimen is modelled as a two-dimensional structure subjected to plane-stress conditions. The glued surfaces between the individual components of the specimen are simulated by means of coherent interfaces. The effect on the failure response by a small geometrical irregularity at the notch tip is studied for the historical specimen dated 1300 A.D. by extending the notch tip with a small, sharp imperfection, see Fig. 13. The size of the imperfection is chosen to be representative of a typical surface irregularity observed at the notch tip of the test specimens.

### Finite element discretization

The geometry of the oak wood specimen is meshed using plane-stress 6-node iso-parametric continuum elements with a 3-point Gauss quadrature. To simulate the discrete fracture behaviour of the specimen, 6-node interface elements equipped with a 3-point Gauss quadrature were placed between all continuum elements, see Fig. 13. This approach was originally proposed in Xu and Needleman (1994) for the simulation of fracture in bulk materials subjected to arbitrary loading conditions, and has been successfully applied to failure analyses of various engineering materials (Cid Alfaro et al. 2010a, b; Tijssens et al. 2001). The number of continuum and interface elements used in the FEM model approximately equals 36,000 and 55,000, respectively. A mesh refinement study not presented here has indicated that this number of elements warrants convergence of the numerical results. The elements were randomly oriented, and the mesh was gradually made finer from the supports towards the central specimen notch in order to adequately describe the local stress concentration evolving around the crack tip.

### Review of interface damage model

For the simulation of discrete fracture, the interface elements in the FEM configuration are endowed with the mixed-mode damage model presented in Cid Alfaro et al. (2009). The main equations of the interface damage model are reviewed below, whereby the general three-dimensional formulation presented in Cid Alfaro et al. (2009) is reduced to a two-dimensional formulation, in correspondence with the plane-stress analyses performed in the current work. Correspondingly, the components of the traction $\mathbf {t}$ and relative displacement $\mathbf {v}$ across an interface are related as

2 $$\begin{aligned} t_i=\left( 1-d\right) K v_{j}-dC_{ij}\delta _{1j}\left<-v_{1}\right>\quad \text {where} \quad i,j\in \{1,2\}, \end{aligned}$$

with the normal and shear directions specified by the indices 1 and 2, respectively. The damage parameter *d* is bounded as $0\le d\le 1$, where $d=0$ corresponds to the initial, undamaged state and $d=1$ represents the completely damaged state. Further, *K* is the elastic stiffness and $\delta _{ij}$ is the Kronecker delta symbol. The penetration of two opposite crack faces is avoided by the second term on the right-hand side of Eq. (2), with the Macauley brackets $\left<x\right>=\frac{1}{2}\left( x+|x|\right)$ warranting that crack faces are in elastic contact when the normal relative displacement $v_1$ becomes negative.

**Fig. 14:**
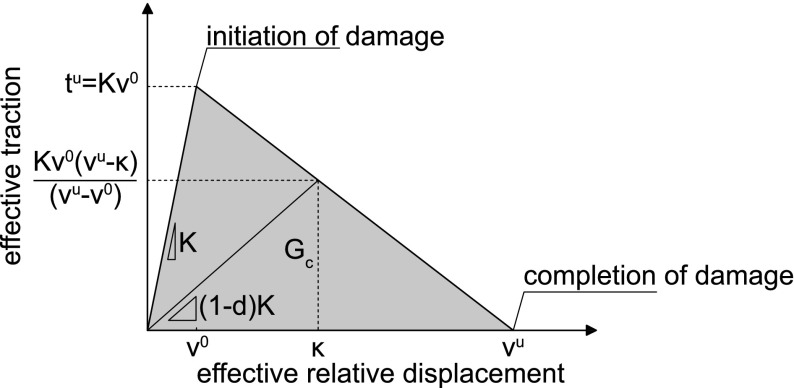
Traction-separation diagram of the interface damage model, taken from Cid Alfaro et al. (2009)

During a loading process, the damage in an interfacial integration point is initiated and evolves with increasing displacement up to the completion of damage. The damage parameter is given by $d=\hat{d}\left( \kappa \right)$, where $\kappa$ is a deformation history variable. The function $\hat{d}\left( \kappa \right)$ is derived from the linear softening curve of the traction-separation law shown in Fig. 14. The initiation of damage is defined by $\kappa =v^{0}$ (corresponding to $d=0$) and completion of damage occurs when $\kappa =v^{u}$ (corresponding to $d=1$), where $v^0$ and $v^u$ are the equivalent crack face displacements at damage initiation and damage completion, respectively. From the linear softening curve shown in Fig. 14, the expression for the damage parameter can be derived as (Cid Alfaro et al. 2009)

3 $$\begin{aligned} d=\hat{d}\left( \kappa \right) =\frac{v^{u}\left( \kappa -v^{0}\right) }{\kappa \left( v^{u}-v^{0}\right) } \, . \end{aligned}$$

The evolution of damage is defined as a rate-dependent process via

4 $$\begin{aligned} \dot{d}= {\left\{ \begin{array}{ll} \begin{aligned} &{}\frac{ \hat{F}\left( \lambda ,\kappa \right) }{\eta }\quad &{}&{}\text {for}\quad \lambda \ge \kappa \quad \text {and}\quad v^{0}\le \kappa< v^{u},\\ &{}0&{}&{}\text {for}\quad 0\le \lambda <\kappa \quad \text {or}\quad \kappa = v^{u}, \end{aligned} \end{array}\right. } \end{aligned}$$

with $\eta$ a relaxation parameter (with dimension of time) and $\hat{F}\left( \lambda ,\kappa \right)$ the damage loading function. The upper expression describes the rate of damage when the effective deformation $\lambda$ exceeds the threshold $\kappa$, while the lower expression sets the rate of damage equal to zero when (i) the threshold value has not (yet) been reached, (ii) the interfacial integration point is in a state of unloading, or (iii) the damage process in the interfacial integration point has completed. The effective deformation is set equal to the Euclidean norm of the vector of relative crack face displacements, $\lambda =|\varvec{v}|=\sqrt{v_{1}^{2}+v_{2}^{2}}$. The loading function is given by

5 $$\begin{aligned} \hat{F}\left( \lambda ,\kappa \right) =\hat{f}\left( \lambda \right) -\hat{d} \left( \kappa \right) =\frac{v^{u}\left( \lambda -v^{0}\right) }{\lambda \left( v^{u}-v^{0}\right) }-\frac{v^{u}\left( \kappa -v^{0}\right) }{\kappa \left( v^{u}-v^{0}\right) }, \end{aligned}$$

where Eq. (3) has been substituted and the form of $\hat{f}\left( \lambda \right)$ has been chosen similar to $\hat{d}\left( \kappa \right)$. In the limit case whereby the relaxation parameter $\eta$ approaches zero, $\eta \rightarrow 0$, Eq. (4) reduces to the rate-independent load condition $\hat{F}\left( \lambda ,\kappa \right) =0$. This case relates to $\lambda =\kappa$, as can be observed from Eq. (5), with the loading–unloading conditions being prescribed by the Kuhn–Tucker relations (Cid Alfaro et al. 2009).

In mixed-mode fracture processes, the relative displacements at damage initiation, $v^{0}$, and damage completion, $v^{u}$, are dependent on a mode-mixity parameter $\beta$, see Cid Alfaro et al. (2009), Turon et al. (2006), which is defined as

6 $$\begin{aligned} \beta = \frac{|v_2|}{|v_2| + \left<v_{1}\right>} \, . \end{aligned}$$

From Eq. (6) it can be observed that pure mode I loading relates to $v_{2}=0$ and thus to $\beta =0$, and pure mode II loading is set by $v_{1}=0$ and thus by $\beta =1$. The functions $v^{0}=\hat{v}^{0}\left( \beta \right)$ and $v^{u}=\hat{v}^{u}\left( \beta \right)$ can be derived based on a mixed-mode failure criterion from linear elastic fracture mechanics (Cid Alfaro et al. 2009; Turon et al. 2006). In this study, a well-known mixed-mode fracture criterion is adopted, which is regularly used to characterize mixed-mode toughness data for brittle interfacial fracture (Hutchinson and Suo 1992; Jensen 1990):

7 $$\begin{aligned} \frac{G_{I}}{G_{I,c}}+\frac{G_{II}}{G_{II,c}} = 1 \, , \end{aligned}$$

where $G_{I}$ and $G_{II}$ are the mode I and mode II energy release rates and $G_{I,c}$ and $G_{II,c}$ are their critical values, as represented by the mode I and mode II fracture toughnesses. Note that Eq. (7) is an extension of the well-known Griffith’s failure criterion, $G=G_{c}$, for brittle materials (Griffith 1921). With the aid of Eqs. (6) and (7), the relative displacements at damage initiation and damage completion can be derived as (Cid Alfaro et al. 2009)

8 $$\begin{aligned} v^{0}=\hat{v}^{0}\left( \beta \right) =v_{1}^{0}v_{2}^{0} \sqrt{\frac{1+2\beta ^{2}-2\beta }{\left( \beta v_{1}^{0}\right) ^{2}+\left( \left( 1-\beta \right) v_{2}^{0}\right) ^{2}}} \, , \end{aligned}$$

and

9 $$\begin{aligned} v^{u}=\hat{v}^{u}\left( \beta \right) =\frac{2\left( 1+2\beta ^{2}-2\beta \right) }{K v^{0}}\left[ \left( \frac{\left( 1-\beta \right) ^{2}}{G_{I,c}}\right) + \left( \frac{\beta ^{2}}{G_{II,c}}\right) \right] ^{-1}, \end{aligned}$$

where $v_{1}^{0}=t_{1}^{u}/K$ and $v_{2}^{0}=t_{2}^{u}/K$ are the relative displacements at which damage is initiated under pure mode I and mode II loading conditions, respectively, and $t_{1}^{u}$ and $t_{2}^{u}$ are the ultimate tractions under pure mode I and mode II loading conditions, respectively.

The numerical update scheme of the above damage model is based on an implicit Backward Euler method and uses a consistent tangent operator for constructing the stiffness matrix. The algorithm is relatively stable and fast, since it does not need numerical iterations, due to the specific damage loading function used, see Eq. (4). The details of the numerical update scheme can be found in Cid Alfaro et al. (2009).

### Dissipation-based path-following method

A dissipation-based path-following method was used to enable the simulation of brittle failure responses with snap-back behaviour, as relevant for the experimental tests discussed in the “Experimental results” section. This method has been presented in Gutiérrez (2004) and can be regarded as an extension of the standard arc-length method originally proposed by Riks (1979). The method has demonstrated to be particularly suitable for the determination of the quasi-static equilibrium path in problems for which crack propagation is the main dissipative phenomenon, and the location of the crack path is a priori unknown (Gutiérrez 2004; Verhoosel et al. 2008). The parametrization of the equilibrium path uses a constraint equation based on the energy release rate of the fracture process, which generally leads to robust numerical results.

### Material properties

As illustrated in Fig. 2, the three-point bending specimens are composed of a central, square block either made of historical oak wood or new oak wood, which is sandwiched by two supporting beams made of new oak wood. The elastic material properties of the historic and new oak wood are summarized in Table 2. The properties were derived from the experimental results presented in the “Experimental results” section, and are in close correspondence with values reported for oak wood in the literature (Reiterer et al. 2002; Saft and Kaliske 2013). It has been validated that the material properties fulfil the Maxwell relations and material stability requirements for orthotropic elastic materials. The effect of material anisotropy on the fracture characteristics is studied for the historic oak wood dated 1300 A.D. by considering two cases: the first case represents an *isotropic* elastic model for which the stiffness modulus of the whole specimen is set equal to that of the T- or R-direction and the Poisson’s ratio is considered to have a representative constant value, while the second case reflects an *orthotropic* elastic model in which the three material directions of oak wood are explicitly accounted for. The strength and toughness properties of the interface damage model are summarized in Table 3, as calibrated from the experimental results presented in the “Experimental results” section. For simplicity, it is assumed that the fracture parameters (tensile strength and toughness) are the same in mode I and mode II, i.e. $t_1^u=t_2^u=t^u, G_\mathrm{I,c}=G_\mathrm{II,c}=G_\mathrm{c}$. Although this assumption may result in an underestimation of the mode II properties, its effect on the failure response generated under 3-point bending, which is dominated by mode I fracture, is small. Note from the linear softening branch depicted in Fig. 14 that the fracture toughness $G_\mathrm{c}$ is defined by the strength $t^u$ and the ultimate crack face separation $v^u$ as $G_\mathrm{c} = t^u v^u/2$. The values of the remaining parameters of the interface damage model were taken similar as in the study reported in Cid Alfaro et al. (2009), which warrant a close correspondence with the rate-independent fracture limit in the interface damage model. It is further emphasized that the value of the elastic dummy stiffness *K* used in the interface damage model was set relatively high, as a result of which the overall elastic response of the three-point bending specimen is not affected by this parameter and is fully determined by the elastic properties of the continuum elements.

**Table 2:** Material properties (elastic moduli *E*, Poisson’s ratios $\nu$, and shear moduli $\mu$) of oak wood

Parameter		Isotropic		Orthotropic		
				Square block		Beam
		TL	RL	TL	RL	LR
*E*	($\text {N/mm}^{2}$)	1120	1600			
$E_{L}$	($\text {N/mm}^{2}$)			12800	12800	12800
$E_{T}$	($\text {N/mm}^{2}$)			$1120^{*}$		
$$E_{R}$$	($$\text {N/mm}^{2}$$)				$$1600^{**}$$	1600
$\nu$	(–)	0.35	0.35			
$\nu _{LT}$	(–)			0.49		
$\nu _{TL}$	(–)			0.04		
$\nu _{LR}$	(–)				0.35	0.35
$\nu _{RL}$	(–)				0.04	0.04
$\mu _{LT}$	($\text {N/mm}^{2}$)			815		
$\mu _{LR}$	($\text {N/mm}^{2}$)				1200	1200

$^{*}$ For the small and large samples made of new oak wood with a TL crack, the tangential elastic modulus was set to $E_\mathrm{T}$ = 500 and 800 $\text {N/mm}^{2}$, respectively

$$^{**}$$ For the historic samples dated 1668 A.D. with an RL crack and the new samples with an RL crack, the radial elastic modulus was set to $$E_\mathrm{R}$$ = 2200 and 1100 $$\text {N/mm}^{2}$$, respectively

## Numerical results

### Failure response and fracture path

**Fig. 15:**
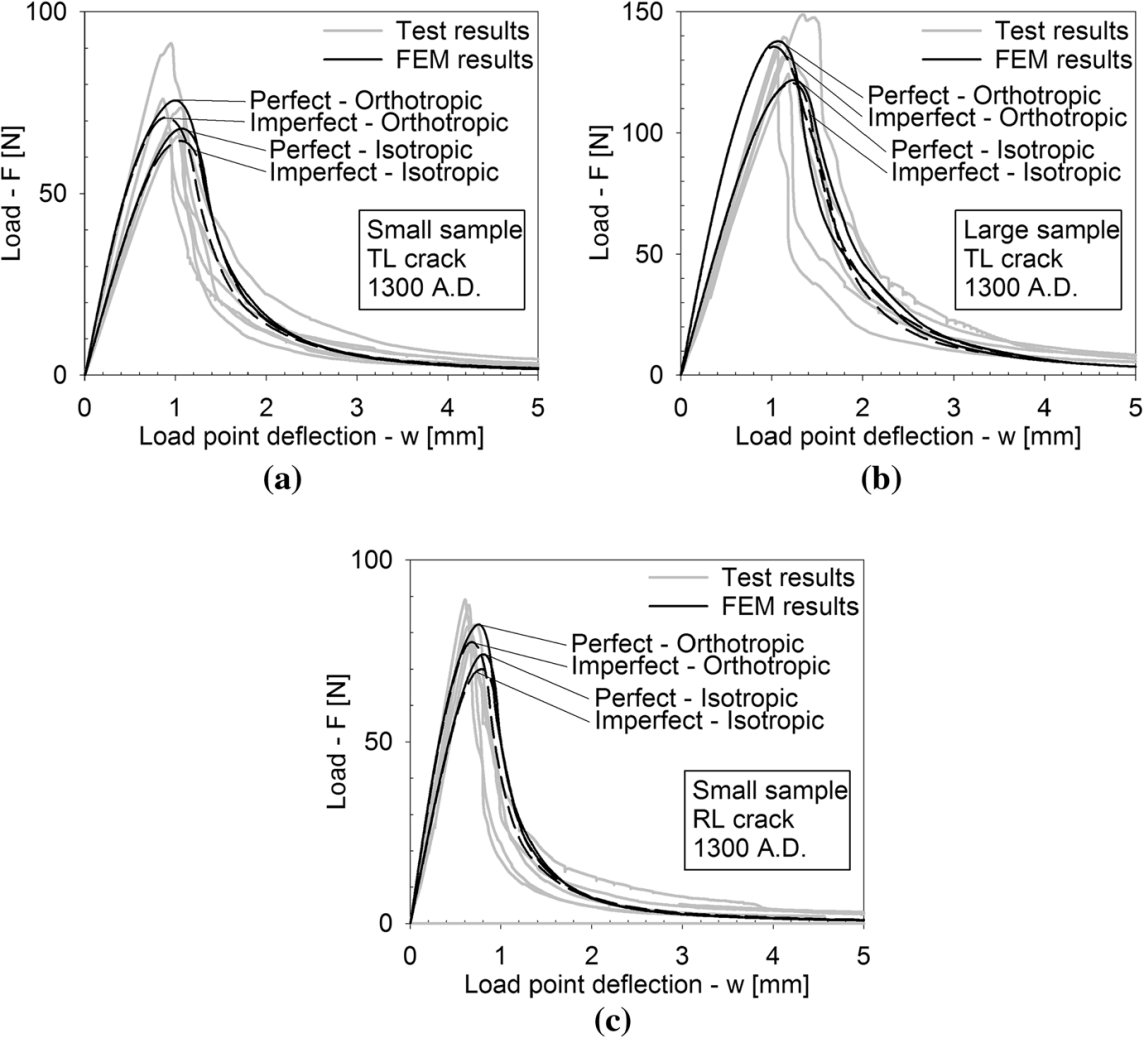
Load–displacement curves for historic oak wooden beams dated 1300 A.D. under three-point bending; FEM result (black line) versus the experimental results taken from Fig. 4 (grey lines). The FEM results are plotted for the isotropic and orthotropic elastic models, with and without an imperfection. **a** Small sample with a TL crack. **b** Large sample with a TL crack. **c** Small sample with an RL crack

**Fig. 16:**
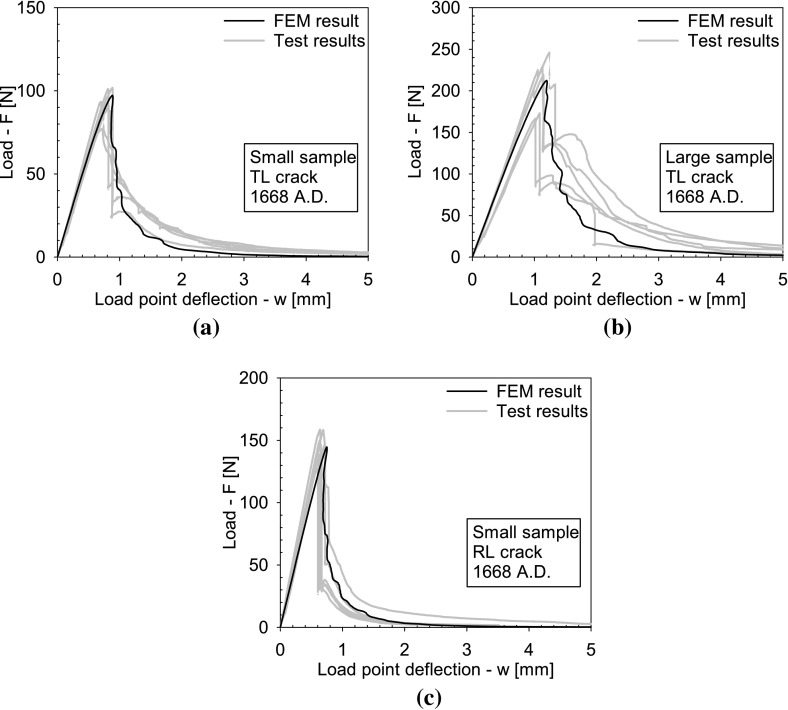
Load–displacement curves for historic oak wooden beams dated 1668 A.D. under three-point bending; FEM result (black line) versus the experimental results taken from Fig. 5 (grey lines). **a** Small sample with a TL crack. **b** Large sample with a TL crack. **c** Small sample with an RL crack

**Fig. 17:**
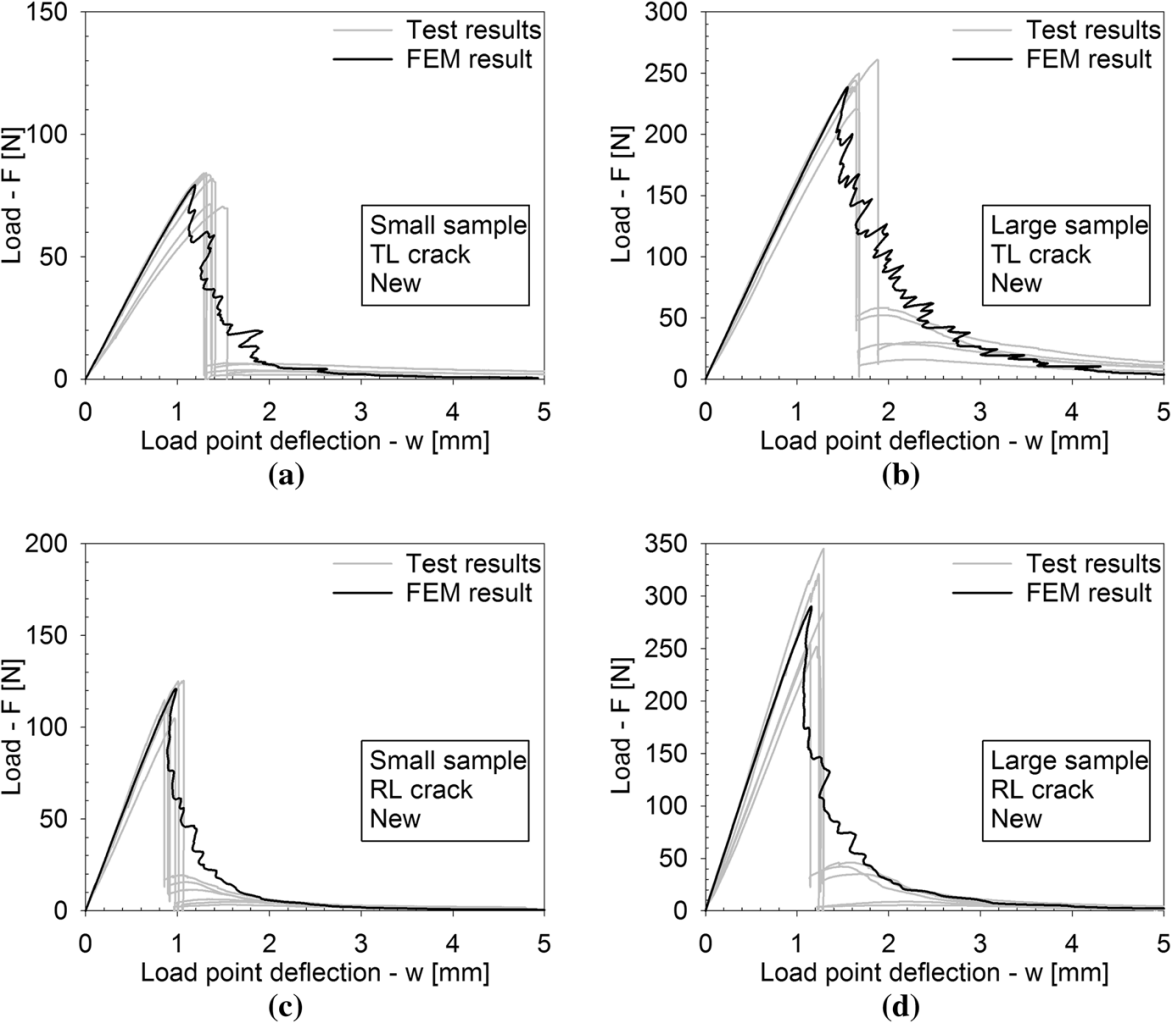
Load–displacement curves for new oak wooden beams under three-point bending; FEM result (black line) versus the experimental results taken from Fig. 6 (grey lines). **a** Small sample with a TL crack. **b** Large sample with a TL crack. **c** Small sample with an RL crack. **d** Large sample with an RL crack

For the historic oak wood specimen dated 1300 A.D, Figure 15 shows the numerical response (black line) together with the experimental response (grey line) taken from Fig. 4. The numerical response is computed both for the isotropic and orthotropic models, thereby distinguishing between specimens with and without an initial imperfection. It appears that the presence of an imperfection at the notch only has a small effect on the failure response, i.e. it decreases the failure load by maximally 7%. Note further that for the orthotropic model the failure load is between 10 and 13% higher than for the isotropic model, and that the response for the orthotropic model in the linear elastic regime is somewhat stiffer. Nonetheless, both the failure load and the fracture energy dissipation for all simulations are in rather close agreement with the experiments, such that it may be concluded that the effect of anisotropy in elastic properties on the overall fracture response is relatively small. In order to reach this level of correspondence, the fracture toughness and tensile strength of the historical oak wood dated 1300 A.D. for the large specimen with a TL crack typically had to be taken smaller than for the small specimen with a TL crack, see Table 3. This difference in fracture properties, however, may *not* be associated with an *energetic size effect* (Bazant 2000), as this effect is explicitly accounted for in the numerical simulations. In specific, the energetic size effect refers to a sample size-dependency of the strength and toughness values caused by stress redistributions in the specimen, as characterized by the interplay between length scales following from the fracture process (i.e. crack length, fracture process zone) and length scales defining the structural geometry (i.e. the specimen dimensions, notch size, imperfection size and location). In the present simulations, the contribution of the relative crack length to the energetic size effect is explicitly included by adopting a *discrete* fracture modelling approach, while the influence by the fracture process zone is accounted for through the softening characteristics employed in the interface damage model. Accordingly, it is concluded that the difference in fracture properties of the large and small specimens should be ascribed to the rather strong inhomogeneities in the wood microstructure, see Fig. 9.

The failure responses from the experimental tests (grey line) and numerical simulations (black line) of the historic oak wood dated 1668 A.D. and new oak wood are depicted in Figs. 16 and 17, respectively. The simulation results refer to an oak wood specimen without an initial imperfection and anisotropic elastic properties. In comparison with the response for the historic oak wood specimen dated 1300 A.D. in Fig. 15, the experimental failure responses obviously are more brittle, as characterized by one or more dynamic drops in the failure load. As explained in the “Experimental results” section, during such a dynamic load drop the quasi-static response is characterized by a snap-back behaviour, which reflects that the energy incrementally dissipated by the brittle failure crack is less than the energy incrementally relased by elastic unloading of the rest of the beam specimen. In the numerical simulations, the quasi-static snap-back behaviour is simulated with the dissipation-based path-following technique described in the “Dissipation-based path-following method” section. The calibration procedure of the numerical model was performed by approximating the value of the peak load and closely matching the *quasi-static parts* of the experimental softening response, during which the load point deflection *w* monotonically *increases* under a *decreasing* load *F*. It can be concluded that this procedure in general leads to an adequate agreement between the experimental and numerical results plotted in Figs. 16 and 17, although for the new specimens the discrepancy on parts of the softening branch appears to be somewhat stronger due to a large drop in the experimental failure load.

**Fig. 18:**
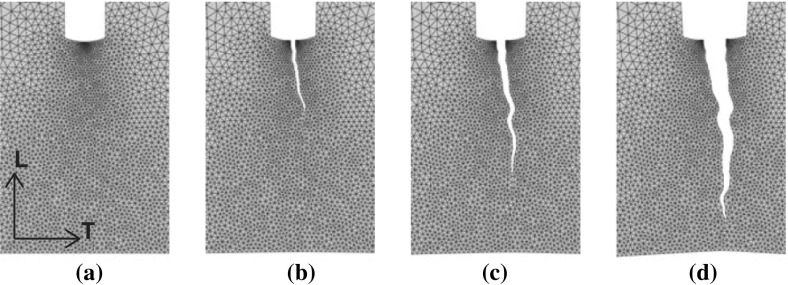
Simulated fracture path of a small orthotropic sample dated 1300 A.D. with a TL crack starting at the geometrical imperfection indicated in Figure 13. **a** Elastic response. **b** Crack initiation. **c** Crack propagation. **d** Ultimate failure

Figure 18 illustrates the simulated fracture path for the TL crack in the small historical sample dated 1300 A.D. A comparison with Fig. 7 shows that this fracture path is in good agreement with the experimental fracture path. Note, however, that there is a small difference in the precise location of crack initiation, which in the numerical simulation is set by a small geometrical imperfection that mimics the effect of a local irregularity present at the notch tip. As pointed out in Fig. 15, the influence of the precise location of the imperfection on the characteristics of the failure response nevertheless is small. For other test specimens, a similar comparison between the numerical and experimental fracture patterns was found; these results are ommitted here for reasons of brevity.

Observe from Figs. 7 and 18 that both in the experiments and the numerical simulations the trajectory of the catastrophic failure crack is characterized by small undulations, due to material heterogeneities and heterogeneities in the finite element mesh, respectively. In order to estimate the effect of such undulations on the energy absorbed during the fracture process, an additional FEM simulation was carried out for the small, historical specimen dated 1300 A.D. and failing by a TL crack, whereby the trajectory of the catastrophic failure crack developing from the notch was enforced to be ideally straight (and thus growing along the vertical specimen direction). Due to the shorter cracking path, the area under the load–displacement curve turned out to be 7% less than for the FEM model with an undulated crack trajectory, from which it may be concluded that the effect of the crack undulations on the energy absorbed is minor.

### Calibration of toughness and tensile strength

**Fig. 19:**
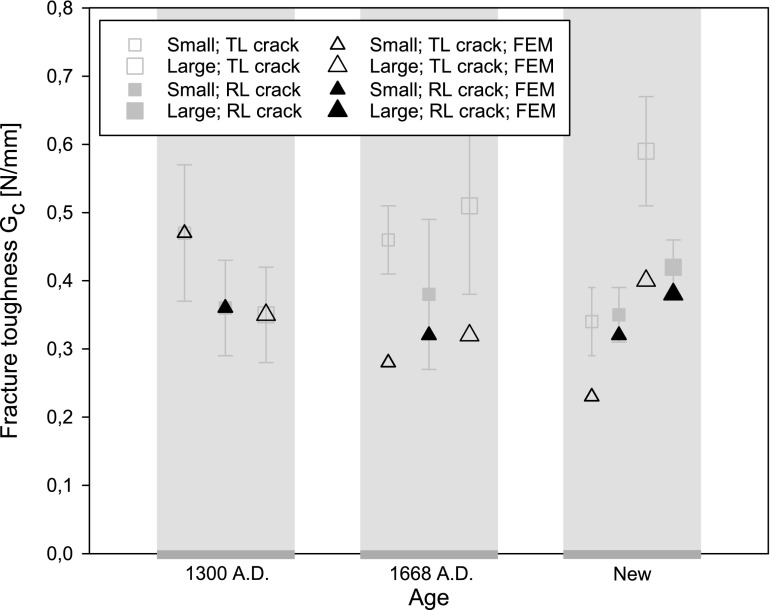
Fracture toughness $G_\mathrm{c}$ as determined from the numerical simulations (black triangles). For reasons of comparison, the average value of $G_\mathrm{c}$ computed according to Eq. (1) is indicated by grey square blocks, where the error bars indicate one standard deviation of uncertainty. The corresponding values are given in Tables 1 and 3

The toughness and strength values calibrated from the FEM simulations of an anisotropic oak wood specimen without an initial imperfection are listed in Table 3. In order to compare these “numerical” toughness values to the “analytical” values computed with Eq. (1), see Table 1, a graphical overview of the toughness parameters is provided in Fig. 19. For the oldest specimen, the numerical toughnesses appear to be very close to the analytical toughnesses, but for the other two specimen ages clearly have a somewhat lower value. This confirms that the dynamic load drops present in the testing of the new specimens and the specimens dated 1668 A.D. cause the area under the experimental failure curve to provide an overprediction of the (quasi-static) fracture toughness. The numerical toughness values do not show a clear dependency on the orientation of the fracture surface with respect to the grain orientation and on the specimen size; the range of values falls between 0.23 and 0.47 N/mm$^1$, which lies somewhat lower than the range reported in the “Oak wood microstructure and fracture toughness” section. for the values determined with Eq. (1). Obviously, this is due to a more accurate modelling of the relatively brittle behaviour of the historical specimens dated 1668 A.D. and the new specimens by the numerical model.

**Table 3:** Fracture toughness $G_\mathrm{c}$ and tensile strength $t^u$ adopted in the interface damage model

Date	Size	Crack orientation	$G_\mathrm{c}$ ($\text {N/mm}^{1}$)	$t^u$ ($\text {N/mm}^{2}$)
Historical 1300 A.D.	Small	TL crack	0.47	5.20
		RL crack	0.36	6.00
	Large	TL crack	0.35	4.00
Historical 1668 A.D.	Small	TL crack	0.28	12.0
		RL crack	0.32	21.0
	Large	TL crack	0.32	12.0
New	Small	TL crack	0.23	18.0
		RL crack	0.32	21.0
	Large	TL crack	0.40	26.0
		RL crack	0.38	21.0

**Fig. 20:**
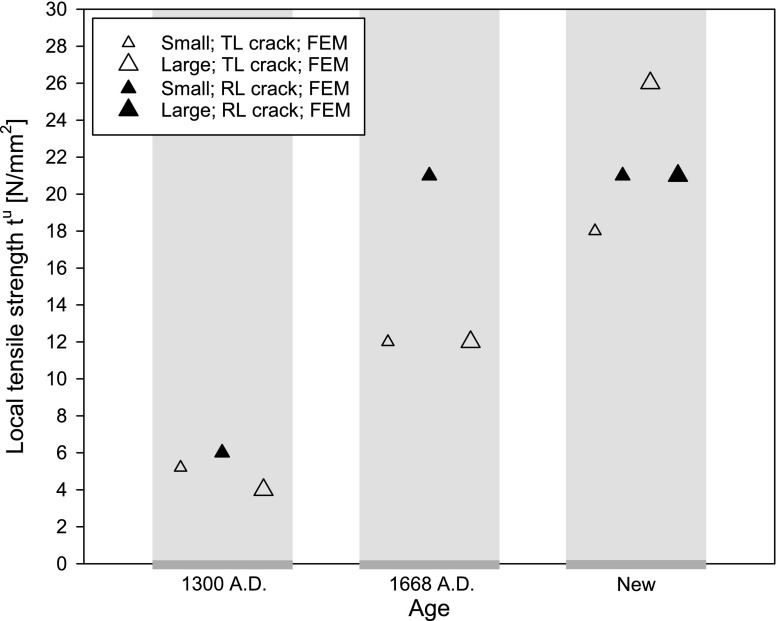
Local tensile strength $t^u$ as determined from the simulations. The corresponding values are given in Table 3

The numerical tensile strength depicted in Fig. 20 for the new specimens generally has the largest value, followed by the historical specimens dated 1668 A.D. and finally the oldest specimens dated 1300 A.D. This decrease in tensile strength may be due to the ageing of oak wood, which, in accordance with Fig. 20, approximately seems to take place as a linear function of time considering that the two time intervals between the three age categories are more or less equal. It can be further observed that for the small specimens the strength related to the RL crack is generally larger than the strength associated with the TL crack. Conversely, the large new specimens show the opposite trend, which therefore does not allow to draw a unique conclusion on this aspect. Finally, it is noted that the strength values for the historical specimens dated 1668 A.D. and the new specimens are a factor 2–3 larger than the values of 6–9 $\text {N/mm}^{2}$ reported for oak wood in the literature (Kollmann and Cote 1968; Saft and Kaliske 2013). A possible reason for this may be that the literature values were determined without considering the effect by the high, local stress concentration emerging at the crack tip. Consequently, the critical stress required for crack initiation turns out lower compared to when this stress concentration is accounted for, as is done in the current FEM analyses.

## Conclusion

The fracture behaviour of oak wood under three-point bending has been studied by experiments and numerical simulations for historical samples dated 1300 A.D., 1668 A.D. and new samples. The main conclusions of this study are summarized below.

(i) For an adequate calibration of the toughness and strength values from a three-point bending experiment, it is necessary to simulate the inhomogeneous stress pattern and failure response in the beam in detail by using an advanced finite element model. By surrounding continuum elements by interface elements equipped with a robust interface damage model, the FEM model allows to accurately describe the complexity of crack initiation and propagation.
(ii) The fracture response of the historical samples dated 1668 A.D. and the new samples investigated in the present communication show a relatively brittle response, leading to dynamic load drops in the quasi-static failure experiments. Under these circumstances, the area under the experimental load-deflection curve no longer serves as an adequate measure for the fracture toughness, but will lead to an overestimation. This effect can be mitigated by accounting for snap-back effects in the quasi-static fracture response, which requires the finite element model of the three-point bending test to be extended with a robust path-following technique. For future work, however, it is worthwhile to investigate the application of test configurations that do not suffer from dynamic load drops during crack propagation, thereby allowing for a straightforward calibration of the fracture toughness of relatively brittle wood samples.
(iii) The effects of wood anisotropy and small imperfections at the notch tip on the failure response of the specimen are relatively small. Due to imperfections, the peak load decreases by maximally 7%. Further, the orthotropic elastic model leads to a failure load that is 10–13% higher than that of a corresponding isotropic elastic model.
(iv) The local tensile strength in the oak wood test specimens decreases with age in an approximately linear fashion. The historical specimens dated 1300 A.D. on average have a local tensile strength of 5 N/mm$^2$, which is about 4 times lower than the average local tensile strength of 21 N/mm$^2$ of the new specimens.
(v) No significant dependence of the fracture toughness on the age of the oak wood has been found. Also, no significant distinction has been measured between the toughness values related to cracking along the TL and RL directions. The measured toughness values generally lie between 0.23 and 0.47 N/mm$^1$, which is in correspondence with the range of values reported in other references (Reiterer et al. 2002; Saft and Kaliske 2013; Vasic and Stanzl-Tschegg 2007).

In future work, the present modelling approach for discrete fracture will be combined with a numerical hygro-thermal model in order to simulate climate-induced damage in decorated oak wooden panels in historical Dutch cabinets and panel paintings. The strength and toughness values measured for the historic and new oak wood will serve as input for this numerical model.

## References

[CR1] Bazant ZP (2000). Size effect. Int J Solids Struct.

[CR3] Cid Alfaro MVC, Suiker ASJ, de Borst R, Remmers JJC (2009). Analysis of fracture and delamination in laminates using 3D numerical modelling. Eng Fract Mech.

[CR2] Cid Alfaro MVC, Suiker ASJ, de Borst R (2010). Transverse failure behaviour of fiber-epoxy systems. J Compos Mater.

[CR4] Cid Alfaro MV, Suiker ASJ, Verhoosel CV, de Borst R (2010). Numerical homogenization of cracking processes in thin fibre-epoxy layers. Eur J Mech A/Solids.

[CR5] Ekelund SE, Jorissen AJM (2014) The museum study of the Climate4Wood research project. In: Jorissen AJM, Leijten AJM (eds) Proceedings of research symposium: structural design with timber, Eindhoven University of Technology, Eindhoven, pp 93–109

[CR6] Ekelund S, Van Duin P, Jorissen A, Ankersmit B, Groves RM (2017) A method for studying climate-related changes in the condition of decorated wooden panels. Stud Conserv, 1–10

[CR7] Griffith AA (1921). The phenomena of rupture and flow in solids. Philos Trans R Soc Lond Ser A.

[CR8] Gutiérrez MA (2004). Energy release control for numerical simulations of failure in quasi-brittle solids. Commun Numer Methods Eng.

[CR9] Hutchinson JW, Suo Z (1992). Mixed mode cracking in layered materials. Adv Appl Mech.

[CR10] ISO (1975) Wood determination of moisture content for physical and mechanical tests., Technical report

[CR11] Jensen HM (1990). Mixed mode interface fracture criteria. Acta Metall Mater.

[CR12] Kollmann FFP, Cote WA (1968). Principles of wood science and technology.

[CR13] Landis EN, Vasic S, Davids WG, Parrod P (2002). Coupled experiments and simulations of microstructural damage in wood. Exp Mech.

[CR14] Lukacevic M, Füssl J (2016). Application of a multisurface discrete crack model for clear wood taking into account the inherent microstructural characteristics of wood cells. Holzforschung.

[CR15] Lukacevic M, Füssl J, Lampert R (2015). Failure mechanisms of clear wood identified at wood cell level by an approach based on the extended finite element method. Eng Fract Mech.

[CR16] Nordtest (1993) Nordtest method, Wood: fracture energy in tension perpendicular to the grain. Technical report

[CR17] Qiu LP, Zhu EC, van de Kuilen JWG (2014). Modeling crack propagation in wood by extended finite element method. Eur J Wood Prod.

[CR18] Reiterer A, Sinn G, Stanzl-Tschegg SE (2002). Fracture characteristics of different wood species under mode I loading perpendicular to the grain. Mater Sci Eng A.

[CR19] Riks E (1979). An incremental approach to the solution of snapping and buckling problems. Int J Solids Struct.

[CR20] Saft S, Kaliske M (2013). A hybrid interface-element for the simulation of moisture-induced cracks in wood. Eng Fract Mech.

[CR21] Tijssens MGA, van der Giessen E, Sluys LJ (2001). Simulation of mode I crack growth in polymers by crazing. Int J Solids Struct.

[CR22] Turon A, Camanho PP, Costa J, Davila CG (2006). A damage model for the simulation of delamination in advanced composites under variable-model loading. Mech Mater.

[CR23] Vasic S, Stanzl-Tschegg SE (2007). Experimental and numerical investigation of wood fracture mechanisms at different humidity levels. Holzforschung.

[CR24] Verhoosel CV, Remmers JJC, Gutiérrez MA (2008). A dissipation-based arc-length method for robust simulation of brittle and ductile failure. Int J Numer Meth Eng.

[CR25] Xu XP, Needleman A (1994). Numerical simulations of fast crack growth in brittle solids. J Mech Phys Solids.

